# VITA Accelerator Neutron Sources: Status and Research Results

**DOI:** 10.3390/cancers18121886

**Published:** 2026-06-09

**Authors:** Sergey Taskaev, Evgenii Berendeev, Marina Bikchurina, Timofey Bykov, Yulia Chesnokova, Rahaf Deeb, Ibrahim Ibrahim, Anna Kasatova, Dmitrii Kasatov, Yaroslav Kolesnikov, Alexey Koshkarev, Ksenya Kuzmina, Victoriia Maltseva, Georgii Ostreinov, Sergey Savinov, Ivan Shchudlo, Stepan Shchukin, Tatiana Shein, Anna Shuklina, Nataliia Singatulina, Evgeniia Sokolova, Igor Sorokin, Iuliia Taskaeva, Gleb Verkhovod

**Affiliations:** 1 Budker Institute of Nuclear Physics, Novosibirsk 630090, Russia; evgeny.berendeev@gmail.com (E.B.); knkstdor@gmail.com (M.B.); timaisabrony@gmail.com (T.B.); jullis74@mail.ru (Y.C.); rahadeeb5@gmail.com (R.D.); ibrahim93za@gmail.com (I.I.); yarullinaai@yahoo.com (A.K.); kasatovd@gmail.com (D.K.); katyono@mail.ru (Y.K.); kent_brockman4@mail.ru (A.K.); k.kuzmina1@g.nsu.ru (K.K.); v.konovalova1@g.nsu.ru (V.M.); wtfsnoo@gmail.com (G.O.); savinov89@gmail.com (S.S.); cshudlo.i.m@gmail.com (I.S.); stepan.shchukin2001@mail.ru (S.S.); sychevatatyanav@gmail.com (T.S.); a.shuklina@g.nsu.ru (A.S.); ntsasht@gmail.com (N.S.); buiya@bk.ru (E.S.); i.n.sorokin@inp.nsk.su (I.S.); inabrite@yandex.ru (I.T.); thevoidscreamer@gmail.com (G.V.); 2 BNCT Laboratory, Faculty of Physics, Novosibirsk State University, Novosibirsk 630090, Russia

**Keywords:** boron neutron capture therapy, neutron source, charged particle accelerator, neutron producing target, dosimetry, beam shaping assembly, boron delivery drug

## Abstract

Purpose: To develop an accelerator neutron source suitable for boron neutron capture therapy—a new promising method for treating malignant tumors—and to develop dosimetry tools and methods. Methods: Research into the transport and acceleration of a beam of charged particles, development and manufacture of an accelerator neutron source, study of the radiation generated, and development and implementation of dosimetry tools and methods. Results: A facility called VITA has been created, which includes a tandem electrostatic accelerator of an original design for producing a 2.3 MeV 10 mA proton beam, a lithium target for generating neutrons in the ^7^Li(p,n)^7^Be reaction, and a beam shaping assembly for forming a therapeutic neutron beam. The facility at the institute is used for scientific research, the facility in Xiamen (China) is used for clinical trials, and the facility in Moscow (Russia) will soon be used for clinical trials. Also, new tools and methods for measuring the boron dose, γ-ray dose, and sum of the fast neutron dose and the nitrogen dose have been proposed and implemented. The conducted studies demonstrated the high efficiency of the VITA^®^ facility, the first possibility of implementing prompt γ-ray spectroscopy for boron imaging, and the first possibility of implementing lithium neutron capture therapy, which has advantages over BNCT, and also presented the results of the development of new tools and methods for measuring the boron dose, γ-ray dose, and the sum of the fast neutron dose and the nitrogen dose. Conclusions: The authors strongly recommend using prompt γ-ray spectroscopy in treatment and developing lithium neutron capture therapy, including in combination with BNCT, and note the high efficiency, reliability and compactness of the VITA^®^ facility.

## Simple Summary

A new method of treating malignant tumors, boron neutron capture therapy (BNCT), is beginning to enter clinical practice. The aim of our study was to develop an accelerator neutron source for this therapy, as well as dosimetry tools and methods. We confirmed the compliance of the developed VITA facility with the presented recommendations, implemented prompt γ-ray spectroscopy for boron imaging, and developed a set of techniques for measuring dose components. We were the first to implement lithium neutron capture therapy, which has advantages over BNCT. Thus, the VITA accelerator neutron source, characterized by high efficiency, reliability and compactness, can be used for BNCT, during which it is recommended to use prompt γ-ray spectroscopy.

## 1. Introduction

Boron neutron capture therapy (BNCT) is considered a promising method for treating malignant tumors [1,2]. Currently, several dozen accelerator neutron source projects are being implemented worldwide, with some of them being used for treatment or clinical trials [2,3,4].

The projects being implemented are distinguished by a wide variety of charged particle accelerators, energy and current of the proton beam and targets for generating neutrons. The following are used as charged particle accelerators: Cyclotrons: 30 MeV 1 mA from Sumitomo Heavy Industries, Ltd. (Japan) [5,6]; 14 MeV 0.4 mA from Heron Neutron Medical Corp. (Taiwan); and 14 MeV 1 mA from the China Institute of Atomic Energy. Radio frequency linacs: 10 MeV 8 mA from DawonMedax (Gyeonggi-do, Republic of Korea) [7]; 8 MeV 5 mA from Mitsubishi (Japan) [8]; 2.5 MeV 12 mA from AccSys Technology, Inc. (Pleasanton, CA, USA) [9]; 3.5 MeV, 4.5 mA and 2.78 MeV 20 mA from the Institute of High Energy Physics (Beijing, China); 2.5 MeV 30 mA from the Lanzhou University (China); and 2.5 MeV, 10 mA in the Xi’an Jiaotong University (China). Electrostatic accelerators: 2.8 MeV 12 mA from IBA (Belgium); 2.6 MeV 30 mA from Neutron Therapeutics (Danvers, MA, USA) [10]; 2.5 MeV, 10 mA in KIRAMS (Seoul, Republic of Korea) [11]; and 1.45 MeV in CNEA (Buenos Aires, Argentina) [12]. It is also worth paying attention to the use of mini neutron generators, as discussed in the review by Leung and Leung (2024) [13]. Detailed information on the projects being implemented, which will allow for comparison, is presented in the IAEA book, 2023 [2]; the Springer book, 2025 [1]; the website of the International Society for Neutron Capture Therapy, 2026 [3]; and in reviews of Zhang et al., 2023 [4]; Dymova et al., 2020 [14]; and Barth et al., 2024 [15]. The most complete discussion of many aspects of BNCT is given in the Springer book, 2025 [1] and in the IAEA book, 2023 [2]. Some important aspects of BNCT are discussed in detail in the articles of Teng et al., 2023 [16]; Zhao et al., 2025 [17]; Chiu et al., 2025 [18]; Dosanjh et al., 2025 [19]; Chen et al., 2024 [20]; Raitano et al., 2023 [21]; Sauerwein et al., 2024 [22]; and Verdera and Praena, 2024 [23].

The task of creating accelerator neutron sources for BNCT, as posed in the 1980s, remained unresolved for a long time. Fortunately, the required parameters have recently been achieved, the most important of which is a high epithermal neutron flux density. The list above indicates that many groups are succeeding in achieving this goal. Among these projects is our VITA neutron source proposed in 1998.

The main objective of the research is to develop a compact accelerator neutron source that meets the requirements of BNCT. The proposed and developed VITA^®^ accelerator neutron source has become in demand for treatment [24] and is actively used for scientific research into various aspects of BNCT.

## 2. Materials and Methods

The VITA^®^ accelerator neutron source is a facility that consists of a tandem electrostatic charged particle accelerator (Vacuum-Insulated Tandem Accelerator; VITA) for producing a stationary monoenergetic proton or deuteron beam with an energy of 2.3 MeV and a current of 10 mA, a lithium target for producing neutrons in the ^7^Li(p,n)^7^Be reaction, and a beam shaping assembly for producing an epithermal neutron beam ([1], pp. 115–125), ([2], pp. 255–260), [25].

BNCT requires neutron beams in the epithermal energy range, ideally in the range of 1 keV to 30 keV [26,27]. The best neutron-generating reaction is considered to be the ^7^Li(p,n)^7^Be reaction due to the rapid increase in the reaction cross-section near the reaction threshold (1.882 MeV) [26,28]. This allows the generation of a sufficient number of relatively low-energy neutrons (200–300 keV) at proton energies of 2.3–2.5 MeV [29]. Such neutrons can be slowed down to the required energy on a relatively thin moderator while maximally maintaining monoenergeticity. The use of a beryllium target, due to its smaller ^9^Be(p,n)^9^B reaction cross-section, requires a higher-energy proton beam. Consequently, the energy of the generated neutrons is higher, requiring a longer moderator, and the energy spectrum of the therapeutic neutron beam will be broader: there will be more undesirable fast neutrons and more undesirable thermal neutrons. The VITA facility was proposed for BNCT with the desire to implement the best solution, and this solution was successfully implemented.

**Figure 1:**
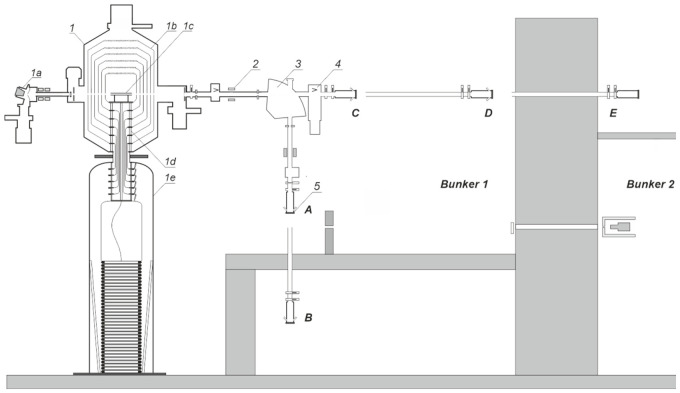
Scheme of the VITA facility: *1*—accelerator (*1a*—negative ion source, *1b*—high-voltage and intermediate electrodes, *1c*—gas stripper, *1d*—feedthrough insulator, *1e*—high voltage power supply), *2*—current transformer, *3*—bending magnet, *4*—retractable Faraday cup, *5*—lithium target (can be placed in one of five positions: *A*, *B*, *C*, *D*, *E*). Adapted from S. Taskaev [25].

At present, the VITA facility at the BINP site looks as shown in Figure 1. This facility makes it possible to obtain not only a proton beam but also a deuteron beam, as well as to generate a neutron flux of various energy ranges. The ion beam is characterized by high monochromaticity (0.1%) and the ability to change energy from 100 keV to 2.3 MeV and current from 0.5 mA to 10 mA. All this allows this facility to be used not only for BNCT but also for other purposes; we will briefly list them at the end of the chapter.

The accelerator *1* consists of a cylindrical vacuum tank with a diameter of 1.4 m and a height of 2.3 m. The openings on the side are for the input and output of the ion beam, on the top for vacuum pumping, and on the bottom for connection to a high-voltage power supply. Inside the vacuum tank, there are five intermediate cylindrical electrodes and high-voltage electrode *1b*. Electrodes are located coaxially with the vacuum tank. Frames for fastening diaphragms are welded into the electrodes on both sides, and diaphragms with an aperture usually 20 mm in diameter are inserted in the negative ion acceleration path and in the high-voltage electrode, and 30 mm in the positive ion acceleration path. The diaphragms are located along the diameter coaxially with the input and output flanges of the vacuum tank and form an accelerating channel. The potential on the high-voltage and intermediate electrodes *1b* is supplied from the high-voltage power supply *1e* through a feedthrough insulator *1d*. A gas stripper *1c* is installed inside the high-voltage electrode coaxially with the accelerating channel, designed to convert negative ions into positive ones.

A surface-plasma source *1a* with a Penning geometry of the gas-discharge chamber is used to produce a beam of negative hydrogen ions, which is focused by a magnetic solenoid onto the accelerator input. At a distance of 57 mm in front of the input of the accelerator, the typical size of the ion beam is 8–9 mm, the convergence is ±30 mrad, and the normalized emittance is from 0.13 mm mrad at a current of 0.5 mA to 0.2 mm mrad at a current of 3 mA.

This focusing of the ion beam at the accelerator’s entrance aperture ensures a “hard” input beam. A highly divergent negative ion beam enters the accelerator, which is focused into a nearly parallel beam 4–5 mm in diameter by the accelerator’s powerful electrostatic input lens. In the gas stripper of the tandem accelerator, negative ions are converted into positive ones. Then, positive ions are accelerated by the electric field and, leaving the accelerator, are slightly defocused by the accelerator’s output electrostatic lens. The proton beam has a transverse size of 10 ± 1 mm, angular divergence from ±0.5 mrad to ±1.2 mrad, and normalized emittance of 0.2 mm mrad at a distance of 1.86 m from the accelerator center.

The lithium target *3* is placed in different positions for different applications. In Figure 1, they are marked as *A*, *B*, *C*, *D*, and *E* positions. A bending magnet *2* is used to direct the ion beam downward.

**Figure 2:**
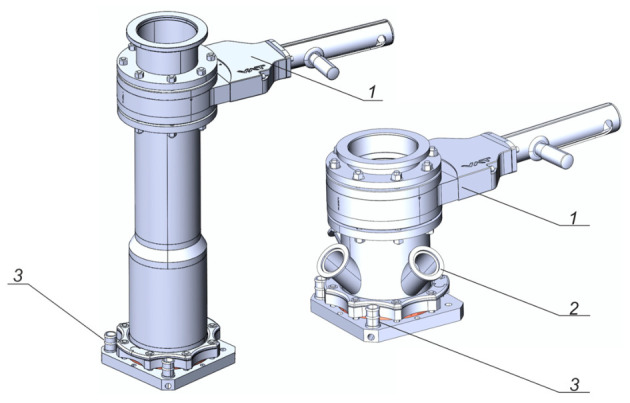
Target units: 1—gate valve, 2—pipes, 3—cooling supply.

The target units (*3* in Figure 1) are made in the form of an aluminum tube with a diameter of 100 mm (Figure 2). Using a gate valve *1* with a standard CF100 connection, the unit is connected to the facility or to a specially designed lithium evaporation unit. For different applications, the target unit varies in length (from 131 mm to 443 mm), and there is the presence or absence of pipes for diagnostic equipment or observation windows located at an angle of 45° or 52.5° to the axis. A copper disk with a thickness of 8 mm is sealed on the end of the target unit. On the proton beam side, a layer of lithium with a diameter of 92 mm and a thickness of 0.5 to 100 μm is applied to the copper disk using thermal evaporation in vacuum. On the reverse side of the copper disk, four double-flow spiral channels are machined for water cooling. A flat aluminum disk with holes for cooling water supply and drainage is pressed against the reverse side of the copper disk. Turbulent water flow in the cooling channels ensures efficient heat removal. It should be noted that the lithium target is characterized by an extremely long service life due to its insensitivity to radiation blistering [30].

For scientific research on BNCT with cell cultures and laboratory animals, as well as for the treatment of pets with spontaneous tumors, the lithium target is placed in position *A*. The placement of a lithium target in position *C* is usually used to measure the parameters of the formed neutron beam in air and in a water phantom using developed diagnostic tools. The results of the conducted research are presented below in Section 3.

A lithium target is placed in position *B* to generate fast neutrons in the Li(d,n) reaction for radiation testing of materials and equipment, including those developed for the International Thermonuclear Experimental Reactor ITER and the Large Hadron Collider at CERN. The article by Bikchurina et al., 2026 [31] provides references to articles containing research results. In the radiation-protected bunker, an additional room was made from concrete blocks with boron carbide, with a wall and ceiling thickness of 46 cm to ensure an acceptable dose level in the control room.

The lithium target is placed in positions *C* or *E* when measuring the yield of particles in nuclear reactions and the cross-section of nuclear reactions. To date, the cross-sections of 21 nuclear reactions have been measured, which are important for BNCT, the generation of a powerful flux of fast neutrons and neutron-free thermonuclear energy. The article by Bikchurina et al., 2026 [31] provides references to articles containing results of measurements of nuclear reaction cross-sections.

A lithium target is placed in position *C* to generate monoenergetic neutrons for the calibration of high-sensitivity two-phase cryogenic avalanche detectors designed to search for dark matter.

A lithium target is placed in position *D* to produce a thin beam of cold neutrons to confirm the solution obtained when solving the quantum problem of neutron motion in a 2(n + 1)-pole magnet (Stern–Gerlach experiment).

Two next-generation VITA-II accelerator neutron sources have been developed for oncology clinics. Three modifications have been made to them [32]. Firstly, the surface plasma source with the Penning geometry of the gas-discharge chamber developed by BINP was replaced by a D-Pace Filament Volume-Cusp Source [33]. Secondly, the negative hydrogen ion beam injected into the accelerator is additionally pre-accelerated by 100 keV. Thirdly, the sectional rectifier (high-voltage power supply) was turned over and placed on the bottom of the feedthrough insulator, which made it possible to significantly reduce the facility height.

**Figure 3:**
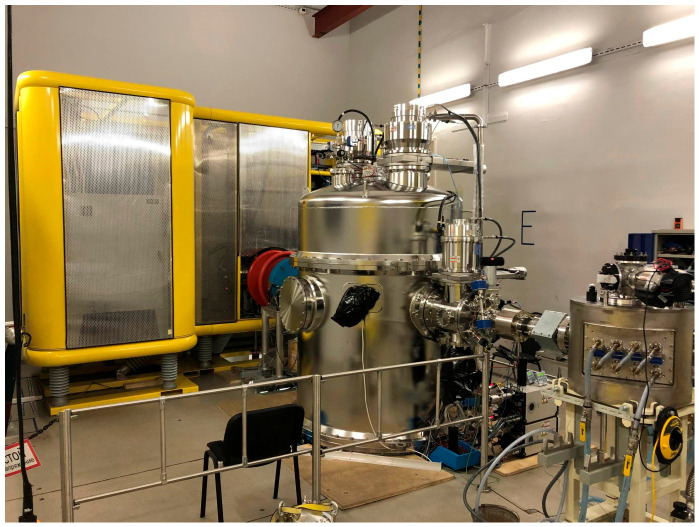
The VITA-IIα facility at the BINP site before being shipped to China.

The VITA-IIα facility was delivered to the BNCT Center at Xiamen Humanity Hospital (Xiamen, China), where clinical trials began on 9 October 2022. A photograph of the facility at the BINP site before being shipped to China is shown in Figure 3. The results of the first patient’s treatment have been published by Hong et al., 2025 [24].

The VITA-IIβ facility was delivered to the Blokhin National Medical Research Center of Oncology of the Ministry of Health of the Russian Federation (Moscow, Russia), where clinical trials will begin in April 2027.

## 3. Results

### 3.1. New Boron Delivery Drugs

Currently, sodium borocaptate (BSH) and boronophenylalanine (BPA) are used for BNCT [34]. The development of new boron delivery drugs is relevant for improving therapy and expanding the range of tumor types that can be treated. A significant number of new drugs have been tested in cell cultures and laboratory animals at the VITA facility. The article by Bikchurina et al., 2026 [31] provides references to 10 articles with the results of testing new boron delivery drugs. One of the drugs was tested in the treatment of a domestic cat, and it showed quite acceptable accumulation [34].

### 3.2. Pet Therapy

Thirty-three pets (cats and dogs) with spontaneous tumors were treated, primarily with BPA, and in six cases with gadolinium. In this case, control groups were not formed since neutron capture therapy was used in these animals with spontaneous tumors as an experimental palliative therapy, given the fact that by the time neutron capture therapy was conducted, all possible treatment options in each specific case had already been exhausted. Pets were not randomized. Irradiation occurred between 1:00 PM and 5:00 PM. The experimental unit was a single animal.

In the first nine cases, BPA was used, and the data obtained indicate a partial tumor response to BNCT. The next seven incurable pets with spontaneous tumors were treated using gadolinium, and there were no significant effects on the life expectancy and quality of life of animals with spontaneous tumors. Following this result, we stopped using gadolinium in therapy. The article by Bikchurina et al., 2026 [31] provides references to articles with the results of pet treatment.

In the treatment of the last 10 pets, the method of prompt γ-ray spectroscopy was used to control the dose and to study the pharmacokinetics of the drug [35]. An hour prior to irradiation, an intravenous infusion of BPA solution with fructose in deionized water was started. The dosage of BPA was 700 mg per kilogram of animal weight, and the solution volume was 20 mL per kilogram of animal weight. In one case, infusion of the drug was continued during irradiation. The primary outcomes of the animal therapy (tumor mass, irradiation parameters, boron concentration in blood) are presented and described in detail in the article by Maltseva et al., 2026 [35].

The methodology of prompt γ-ray spectroscopy and ethical approval were as detailed in [35]. The study was approved by the Institutional Review Board (protocol #91, 5 October 2021) in compliance with European Community Directive 86/609/EEC. Due to the palliative nature of the treatment (all animals arrived in severe or critical condition with advanced age), no control group was formed—this was a descriptive case series. Statistical analysis was descriptive: inter-subject variability for the 478 keV line intensity (boron neutron capture reaction count) was 10-fold (range 13,000–130,000 counts, on average 50,000 ± 35,000 counts), and its e-folding reduction time (boron clearance from tumor) was 6-fold (range 3–17 h, on average 8 ± 4 h). No correlation was found between capture intensity and tumor volume or blood boron concentration. For the last animal (case No. 10), follow-up X-ray at one month after therapy showed a 26-fold tumor volume reduction (from 12.7 g to 0.48 g) [23]. For the other animals, clinical assessment (not systematic imaging) indicated tumor reduction and improved condition, as noted in [35].

Due to the palliative setting and the fact that many owners opted for euthanasia shortly after treatment (driven by the animals’ initial severe condition, advanced age, or financial constraints rather than tumor progression), reliable median survival times could not be calculated. Assessment of acute toxicities was not systematically performed. Tumor reduction and clinical improvement were observed [35]. No severe adverse effects requiring treatment interruption were noted.

### 3.3. Dosimetry

Dosimetry in BNCT is fundamentally more complex than in other radiation therapy methods. This is because BNCT typically considers four dose components with different RBE: boron dose, nitrogen dose (thermal neutron dose), fast neutron dose, and γ-ray dose [1]. “*The first two dose components cannot be measured in principle*”, as previously written in ([36], p. 279). There are also no instruments for measuring the fast neutron doses, as all certified dosimeters are calibrated to the harder neutron spectrum. Only methods and instruments are available for measuring the γ-ray dose.

#### 3.3.1. Prompt γ-Ray Spectroscopy

The neutron capture reaction by boron itself provides a direct measurement opportunity since in 93.9% of cases, one of the reaction products is the emission of a 0.478 MeV γ-quantum. The number of nuclear reactions that have occurred in the observed volume can be determined by measuring the intensity of radiation of photons with an energy of 478 keV. This method of measurement in relation to BNCT has been proposed and described by Kobayashi and Kanda, 1983 [37]; it is called prompt γ-ray spectroscopy. Although this method is well-known, it is practically not implemented. The difficulty in implementing the method is that the γ-ray spectrometer with good energy resolution must be placed in the neutron flux. It must also be taken into account that photons of the same energy are also emitted from the lithium target due to inelastic scattering of protons on lithium nuclei. If a γ-spectrometer that is relatively resistant to neutron flux is used, its energy resolution will not allow the separation of the 478 keV line from the more intense 511 keV line. An HPGe γ-ray spectrometer separates these lines but is not resistant to neutrons. Efforts to introduce this method have been made for many years but have been unsuccessful.

To implement this method of prompt γ-ray spectroscopy, we placed the γ-ray spectrometer as far as possible from the irradiation zone and protected it from neutrons as much as possible. We were lucky that the bunker housing the neutron source was adjacent to another bunker (bunker 2, see Figure 1); the spectrometer was placed in it, behind a 1.5 m concrete wall. A hole was drilled in the concrete wall to register radiation from the irradiation object. A collimator made of lead bricks was placed in front of the pet. A plexiglass plate, providing neutron scattering, was installed in front of the wall hole in bunker 1 to suppress neutron penetration into bunker 2. A similar plexiglass plate was installed in bunker 2 in front of the spectrometer. The spectrometer detector was covered in cadmium foil to absorb thermal neutrons to ensure better safety of the spectrometer. The spectrometer detector was placed inside a lead collimator to reduce background signal and detector load. Measurements were performed using the SEG-1KP HPGe γ-spectrometer (Institute of Physics and Technology, Dubna) based on a semiconductor detector made of high-purity germanium.

**Figure 4:**
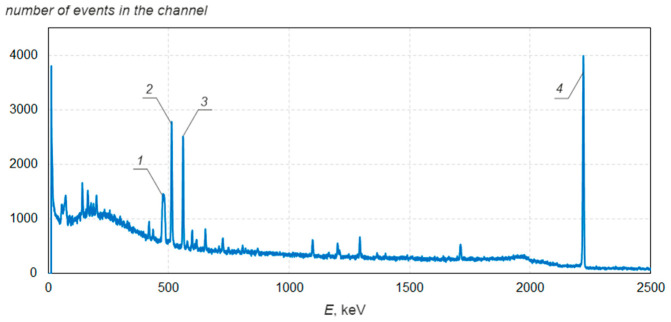
Characteristic energy spectrum of γ-ray measured by the HPGe γ-spectrometer: *1*—478 keV, *2*—511 keV, *3*– 517 keV, *4*—2223 keV.

The first application of prompt γ-ray spectroscopy to evaluate boron uptake and clearance during BNCT was performed on a cohort of ten pets by Maltseva et al., 2026 [35]. A typical spectrum of the detected radiation is shown in Figure 4. It is quite clear that the 478 keV photon line is broadened due to the Doppler effect, since the photon emission occurs from a still-flying lithium-7 nucleus. Shielding the lithium target with lead bricks and significantly suppressing the neutron flux by placing plexiglass scatterers and cadmium foil made it possible to almost completely suppress 478 keV photons from the lithium target in the reaction of inelastic scattering of protons on lithium atomic nuclei and ensure the registration of 478 keV photons only from the reaction of neutron absorption by boron. Other lines in the measured spectrum are noteworthy. The 511 keV line is associated with annihilation processes; the 517 keV line is associated with neutron capture by chlorine in the ^35^Cl(n,γ)^36^Cl reaction, and its intensity is proportional to the volume of tissue in the detection region; the 558 keV line is associated with neutron capture by the cadmium foil in the ^113^Cd(n,γ)^114^Cd reaction, and its intensity is proportional to the number of neutrons scattered from the detection region; and the 2223 keV line is the line emitted during neutron capture by hydrogen in the ^1^H(n,γ)^2^H reaction, and its intensity is also proportional to the volume of tissue in the detection region.

**Figure 5:**
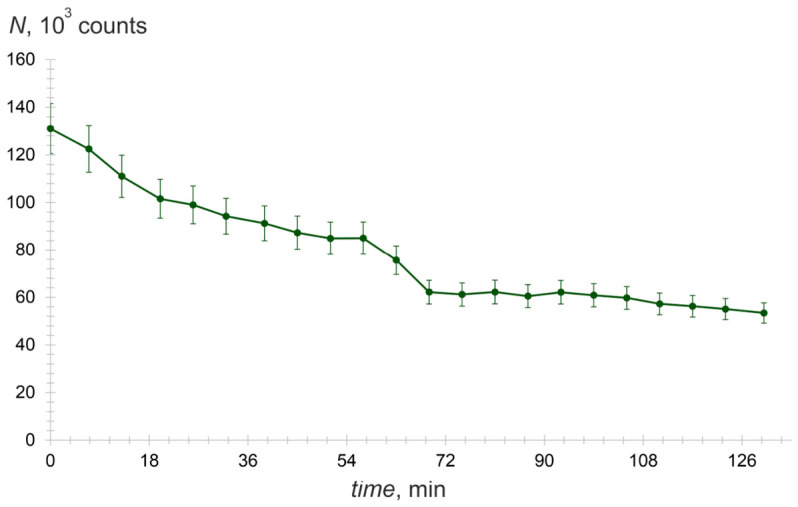
Time dynamics of the 478 keV line event count *N* (normalized to a proton fluence of 1 mA·h) during one of the irradiations.

Figure 5 presents the time dependence of the 478 keV line event count during one of the irradiations. Among all irradiated pets, the highest photon yield was recorded for this cat with adenocarcinoma in the nasal cavity, which was characterized by rapid tumor growth, indicating the greatest boron accumulation in the tumor. The graph also shows a rapid decline in boron accumulation; the signal reduction time by a factor of *e* is 3 h. By moving the collimator and monitoring the 478 keV, 511 keV, 558 keV, and 2.223 MeV lines, data can be obtained indicating the selectivity of boron accumulation in the tumor.

The studies revealed that the intensity of nuclear reactions of neutron capture by boron in different pets differs by 10 times, and the boron clearance time differs by 6 times. This result was extremely unexpected. There was no correlation between the measured intensity of boron neutron capture events and the boron concentration in the tumor or blood. Attention should also be drawn to the differing dynamics of boron clearance from the tumor (ranging from 3 to 17 h) and from the blood (ranging from 1 to 3 h).

There are many potential factors that can cause interindividual variability in boron dynamics. To clarify this, we plan to conduct additional studies in 10 pets, limiting the variety of tumors and restricting the study design. We plan to treat pets with adenocarcinoma and use a mobile collimator to determine the time dynamics of boron accumulation in the tumor, in healthy tissue, and in the kidneys. We will also try to evaluate the therapeutic effect using ongoing assessment of animal condition.

The obtained data indicate that for therapy planning and outcome assessment, prompt γ-ray spectroscopy is recommended for the treatment of patients using BNCT.

#### 3.3.2. Compact Neutron Detector

A compact neutron detector has been developed and manufactured to measure the boron dose and γ-ray dose. The detector’s sensors have an identical design, each containing three independent optical detection channels. The first detection channel is based on a cast polystyrene scintillator with boron, the second is based on a similar scintillator without boron, and the third is an optical fiber without a scintillator. The scintillators are cylindrical, 1 mm in diameter and 1 mm long. The scintillators are mounted on the ends of the optical fiber using optical organosilicon rubber and protected by a light-tight plastic housing. The sides of the scintillator, as well as the end of the third optical fiber, are coated with a reflective coating. Light from the scintillators is transmitted via optical fibers to the readout electronics, and optical pulses are detected using micropixel avalanche photodiodes (SiPM). The number of registered events with an amplitude above a certain threshold at 10 ms intervals is transmitted to the computer for further processing. The difference between the counts of the two detectors, with and without boron, allows us to estimate the contribution of the neutron component recorded by the detector. The difference in the counts in the detector channel without boron and the number of events recorded in the channel without a scintillator produces a signal proportional to the γ-ray dose at the measurement point, since it eliminates the contribution of Cherenkov radiation generated in the optical fiber.

Using a compact neutron detector, we measured the spatial distribution of boron dose rate and γ-ray dose rate in air and in a water phantom for several variations of the neutron beam shaping assembly during its optimization. The article by Bikchurina et al., 2026 [31] provides references to articles with the results of the development and use of the compact neutron detector.

#### 3.3.3. Cell Dosimeter

The use of a lithium target allows for a new method of measuring the sum of fast neutron dose and nitrogen dose (thermal neutron dose). The idea is that at proton beam energies below the neutron generation threshold of 1.882 MeV, cell cultures can be irradiated with γ-radiation alone as a result of the inelastic scattering of protons on lithium nuclei (the ^7^Li(p,p’γ)^7^Li reaction). At proton beam energies above the neutron generation threshold of 1.882 MeV, cell cultures placed in the same location are irradiated with mixed radiation: neutrons and γ-radiation. If the proton beam current is reduced to a certain value, then the same biological effect can be achieved: for example, the survival of cell cultures by irradiating them with two different types of ionization for the same time. If irradiating cell cultures of the same line with two different types of radiation for the same duration results in equal cell survival, then, by definition, the equivalent doses are equal. To implement this method, it is sufficient to measure the γ-ray dose *D*_γ_, for which there are many measuring instruments. Hence, the sum of the fast neutron dose and the nitrogen dose *D*_n_ is calculated by the formula *D*_n_ = *D*_γ standard_ − *D*_γ mixed_, where *D*_γ standard_—the γ-ray dose when the cells are exposed to γ-radiation; and *D*_γ mixed_—the γ-ray dose when the cells are exposed to mixed radiation.

This new dosimetry method has been experimentally tested by Dymova et al., 2021 [38]. The glioblastoma cell line U251 was used. Equal cell line survival of 34 ± 4% was achieved when the proton beam parameters of 1.5 h irradiation were as follows: in the γ-radiation mode—1.800 ± 0.002 MeV, 2.17 ± 0.03 mA; and in mixed radiation mode—2.050 ± 0.002 MeV, 1.40 ± 0.05 mA. The γ-dosimeter DBG-S11D (Doza LLC, Moscow, Russia) measured the dose received by the cells in both cases. In the γ-radiation mode, cells received 5.21 Gy, and in the mixed radiation mode—3.98 Gy. Therefore, the sum of fast neutron dose and nitrogen dose was 1.23 Gy-Eq.

Please note that existing fission ionization chambers and dosimeters with a moderator are not applicable since calibration with neutrons with a harder spectrum, usually 2 MeV, will overestimate the dose readings by several times.

#### 3.3.4. Epithermal Neutron Flux Monitor

An epithermal neutron flux monitor, which is an activation detector using the ^71^Ga(n,γ)^72^Ga reaction, is proposed for BNCT by Guan et al., 2015 [39]. The ^71^Ga(n,γ)^72^Ga reaction is characterized by many resonances, and by placing the activated material (gallium) inside the moderator, the same sensitivity to neutrons of different energies can be achieved. In the monitor, the activation material is positioned in the center of the polymethyl methacrylate (PMMA) or high-density polyethylene (HDPE) cylinder and covered with cadmium foil for thermal neutron absorption. Numerical neutron transport simulation shows that the detector is sensitive to epithermal neutrons, and it has a flat sensitivity curve in the epithermal neutron range, while its sensitivity to thermal and fast neutrons is low. It was experimentally measured that the sensitivity of the flux monitor with an HDPE moderator is 1.24 times higher than the sensitivity of the flux monitor with a PMMA moderator. It is also proposed to equip the flux monitor with a titanium disk, which further reduces the detector’s sensitivity to fast neutrons. The article by Bikchurina et al., 2026 [31] provides references to articles with the results of the development and use of the epithermal neutron flux monitor.

There is a problem with the available data on the ^71^Ga(n,γ)^72^Ga reaction cross-section. While in the resonance region, the reaction cross-sections are practically the same in the ENDF-VII and JENDL-4.0 libraries, and in the energy region below the resonances (below 50 eV), the cross-section in the ENDF-VII library is approximately 1.3 times larger than that in the JENDL-4.0 library. It is necessary to determine which of the used cross-sections is reliable for practical use of the flux monitor.

#### 3.3.5. Labeling of the Boron Delivery Drug with Activated Nuclei

The idea is to label the boron delivery drug with a stable atomic nucleus characterized by a large neutron capture cross-section. During irradiation, these atomic nuclei will become radioactive, and their spatial distribution can be measured with a γ-ray spectrometer after therapy. Nuclei such as ^109^Ag, ^115^In, and ^197^Au are considered candidates. An approach to assessing the absorbed dose of BNCT using gold nanoparticles has demonstrated efficacy and safety in a cell culture experiment. The article by Bikchurina et al., 2026 [31] provides a reference to an article with this proposal.

### 3.4. Fundamental Knowledge

When using an accelerator neutron source for treating patients, validation of the lithium target is required, including measurement of the neutron yield from the lithium target in the ^7^Li(p,n)^7^Be reaction. Such validation was carried out. The neutron yield from a lithium target was measured by its activation with the radioactive isotope beryllium-7 using the HPGe γ-ray spectrometer. The measured yield is shown to correspond to that calculated by Lee and Zhou, 1999 [29], with an accuracy of 5%, which is important for planning the treatment. The article by Bikchurina et al., 2026 [31] provides a reference to an article reporting the results of measuring the neutron yield from the lithium target.

The use of a lithium target entails additional emission of 478 keV photons as a result of inelastic scattering of a proton by lithium nuclei (^7^Li(p,p’γ)^7^Li reaction). The accompanying photon absorbed dose is undesirable for BNCT. Knowing the photon yield of the ^7^Li(p,p’γ)^7^Li reaction is certainly important for nuclear data evaluation and for estimating the absorbed dose when planning therapy. However, the data of the 478 keV photon yield and the data of the ^7^Li(p,p’γ)^7^Li reaction cross-section are limited and differ significantly. We measured with high accuracy the ^7^Li(p,p’γ)^7^Li reaction cross-section and 478 keV photon yield from a thick lithium target at proton energies from 0.65 MeV to 2.225 MeV. The article by Bikchurina et al., 2026 [31] provides a reference to an article reporting the results of measuring the 478 keV photon yield and the cross-section of the ^7^Li(p,p’γ)^7^Li reaction, as well as references to earlier articles. The data we received in 2021 can help establish a benchmark for radiation protection and treatment planning. It is important to note that the results of measurements subsequently carried out by a Greek group in 2023 [40] and an Iranian group in 2026 [41] coincide with those we measured with good accuracy. The results recently obtained by us, as well as the Greek and Iranian groups, make it possible to take this radiation into account in the absorbed dose with the necessary accuracy.

### 3.5. Beam Shaping Assembly

**Figure 6:**
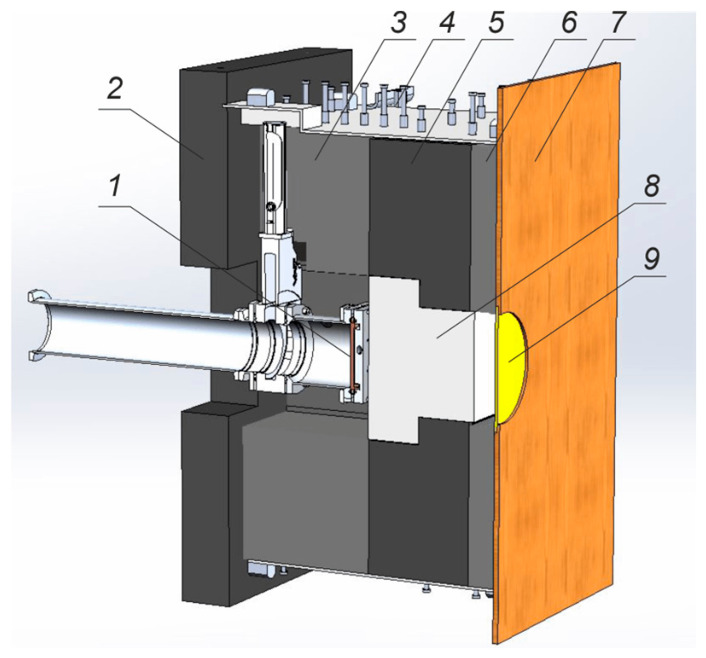
BSA: *1*—lithium target, *2*—curtains made of borated polyethylene, *3* and *6*—lead reflector, *4*—titanium frame, *5*—reflector made of reactor graphite, *7*—bismuth sheet, *8*—moderator made of magnesium fluoride crystals, *9*—filters.

The neutron beam shaping assembly (BSA) was optimized using numerical modeling of neutron and γ-radiation transport. The optimal range of neutron and proton energies, as well as the size and material of the moderator and reflector, were determined. The proton energy was 2.3 MeV, the moderator material was magnesium fluoride crystals, and the reflector material was graphite in the forward hemisphere and lead in the rear hemisphere. Using a compact neutron detector, the calculations were verified, showing good agreement between the calculated and measured data. A view of the BSA for a Moscow clinic is shown in Figure 6. The neutron beam at the 2.3 MeV 7 mA proton beam satisfies all IAEA requirements [2]: therapeutic epithermal flux is 5.6 × 10^8^ cm^−2^ c^−1^, thermal to epithermal ratio is 0.017, fast neutron dose per unit epithermal fluence is 6.5 × 10^−13^ Gy cm^−2^, and γ-ray dose per unit epithermal fluence is 2.0 × 10^−13^ Gy cm^−2^.

Green et al., 2025 [42] presented the depth distribution of the thermal neutron fluence rate, which is proportional to the boron dose rate, for accelerator neutron sources used for treatment or clinical trials. The data presented in this article allow us to compare the BSA we developed with similar ones.

**Figure 7:**
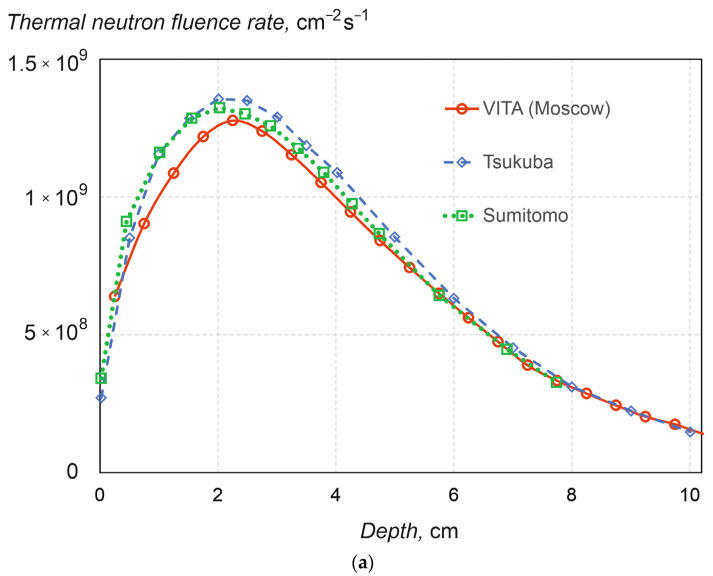

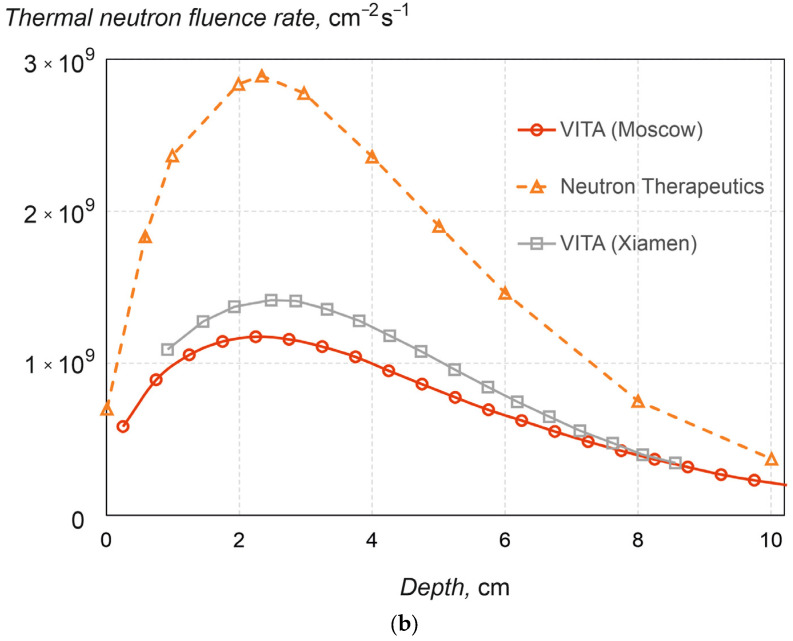
Depth distribution of thermal neutron fluence rate in water (**a**) and in PMMA (**b**).

Figure 7a shows the data on the depth distribution of the thermal neutron fluence rate in water, and Figure 7b shows it in PMMA. The legends in the figures mean the following: “VITA (Moscow)” is a 2.3 MeV 7 mA VITA-IIβ facility manufactured and delivered to Moscow (Russia) for clinical trials; “Tsukuba” is an 8 MeV 4 mA radiofrequency accelerator with a beryllium target used in Tokai (Japan) for clinical trials; “Sumitomo” is a 30 MeV 1 mA cyclotron with beryllium target used in Osaka and Koriyama (Japan) for treatment; “Neutron Therapeutics” is a 2.6 MeV 30 mA single-ended accelerator used in Helsinki (Finland) for clinical trials; and “VITA (Xiamen)” is a 2.35 MeV 10 mA VITA-IIα facility manufactured and delivered to the hospital in Xiamen (China) used for clinical trials.

It is clear that the neutron beam we have formed allows us to obtain a similar deep distribution of the thermal neutron fluence rate and, consequently, the boron dose rate.

If we compare the facilities by efficiency (the ratio of the thermal neutron fluence rate to the proton beam power), we find that the VITA-IIβ facility is the most efficient: it is 2 times more efficient than the “Neutron Therapeutics” facility, 1.8 times more efficient than the “Tsukuba” facility, 1.75 times more efficient than the “Sumitomo” facility, and 1.2 times more efficient than the VITA-IIα facility. The high efficiency is due to the use of a ^7^Li(p,n)^7^Be reaction to generate neutrons, optimization of the lithium target and the use of magnesium fluoride crystals.

For scientific research with cell cultures and laboratory animals, the PMMA moderator is used at the VITA facility; since 2024, an HDPE moderator with a volumetric inclusion of bismuth has been used, the use of which has made it possible to halve the γ-ray dose while maintaining the boron dose.

### 3.6. Treatment Planning System

The VITA treatment planning system (TPS) is designed for therapy planning. VITA TPS is indispensable software for BNCT: it simulates patient exposure using a three-dimensional model made of computed tomography (CT) data and provides calculation and analysis of dose distribution relationships that can be used to select optimal parameters for the therapy. The VITA TPS software package consists of several modules: operator interface, geometry construction utility, particle transport simulation code NMC developed by Yurov et al., 2012 [43], ENDF-VII library of evaluated incident-neutron data, and gRPC server. VITA TPS has a client–server architecture, providing simultaneous operation of several workstations.

VITA TPS interface is represented by a desktop application written in the C# programming language. The interface allows processing CT data with subsequent creation of a voxel model of the patient, which is further imported into the geometry construction utility of the particle transport simulation code NMC. The utility takes the voxel model and irradiation parameters set in a separate configuration file as input. After building the geometry in a format that the particle transport modeling code accepts as an input, the program calculates the dose distributions in the model. The obtained data on the dose component distributions are transferred to the VITA TPS interface, where they are combined with the original tomographic images of the patient and displayed as an isodose map.

Based on the overlap of the dose distributions and the specified contours of the regions of interest, the program output is a dose–volume histogram, which is further interpreted by specialists to decide on the possibility of BNCT. The patient data and irradiation plans are stored on the server to provide centralized data storage. The remote procedure call system (gRPC) is designed for communication between the client and server parts.

The output of TPS VITA is the exposure plan protocol, which is a static representation of the exposure plan. The protocol includes longitudinal and transverse sections with the superimposed contours and the isodose map, and it contains statistics of the dose distribution in the contours. The standard approach to experimental validation of exposure plans is as follows: the patient’s exposure conditions are transferred without changing any parameters to an available phantom using radiation detectors, then the phantom is irradiated, and the measured results are compared with the calculated ones. If the results of measurements and predictions for the phantom coincide, the plan is considered to be correctly calculated, and the patient can be treated.

The calculation results were verified on the VITA facility in experiments with the measurement of boron dose and γ-ray dose in a water phantom for three different BSAs: with a moderator made of magnesium fluoride crystals, with a moderator made of HDPE, and without a moderator. The study results demonstrated good agreement between the measured and modeled boron dose and γ-ray dose depth distributions for all cases considered. In all experiments, the maximum deviation between the calculated and experimental data for boron and gamma doses was comparable to the statistical error.

In Section 3.3.1, we showed the possibility of using the prompt γ-ray spectroscopy method for BNCT, which gives very important and reliable results. We recommend using this method of boron imaging in patient therapy. We have included the following additional features in the VITA TPS: The number of neutron capture reactions by boron-10 ^10^B(n,αγ)^7^Li and the number of neutron capture reactions by hydrogen ^1^H(n,γ)^2^D within the field of view are calculated. The attenuation coefficient for the 478 keV photon flux in the direction of the γ-spectrometer and the attenuation coefficient for the 2.223 MeV photon flux in the direction of the γ-spectrometer are also calculated. Comparison of calculated and measured quantities of nuclear neutron absorption reactions by hydrogen, accounting for the attenuation of the 2.223 MeV photon flux in the direction of the γ-spectrometer placement, provides reliable verification of the neutron flux. Similarly, comparison of the calculated and measured numbers of nuclear neutron capture reactions by boron, accounting for the attenuation of the 478 keV photon flux in the direction of the γ-spectrometer placement, provides reliable determination of the boron concentration. This is important for treatment planning, determining the treatment duration, and evaluating the outcome.

We propose using a lithium target as a radionuclide source of 478 keV photons for the calibration of the γ-spectrometer. The radioactive isotope beryllium-7 is produced in the target upon irradiation with a proton beam *via* the nuclear reaction ^7^Li(p,n)^7^Be. The resulting beryllium nuclei decay with a half-life of 53.22 days, emitting a 478 keV photon. The photon energy from this radionuclide source matches the energy of photons detected by the γ-spectrometer for boron dose measurement. Furthermore, the intensity of this photon source is comparable to the intensity of the emission measured by the γ-spectrometer for boron dose measurement.

### 3.7. VITA-III Facility

Let us recall that the second version of the VITA facility uses pre-acceleration of the beam of negative hydrogen ions injected into the accelerator.

**Figure 8:**
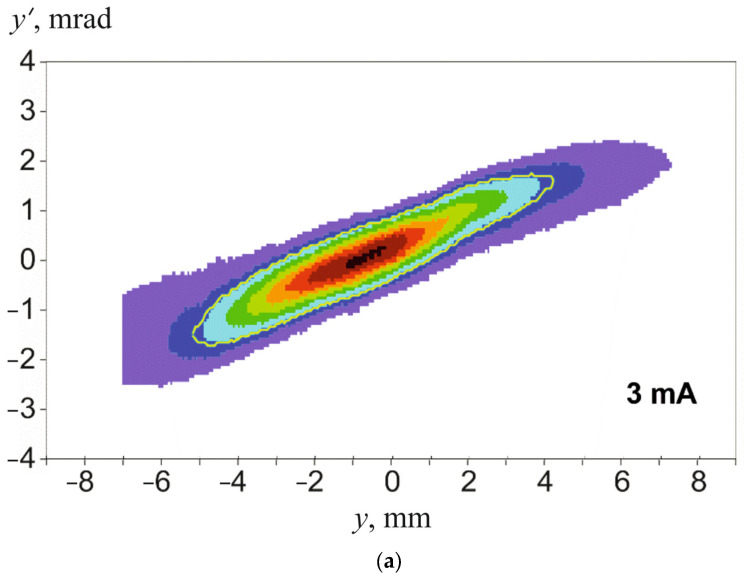

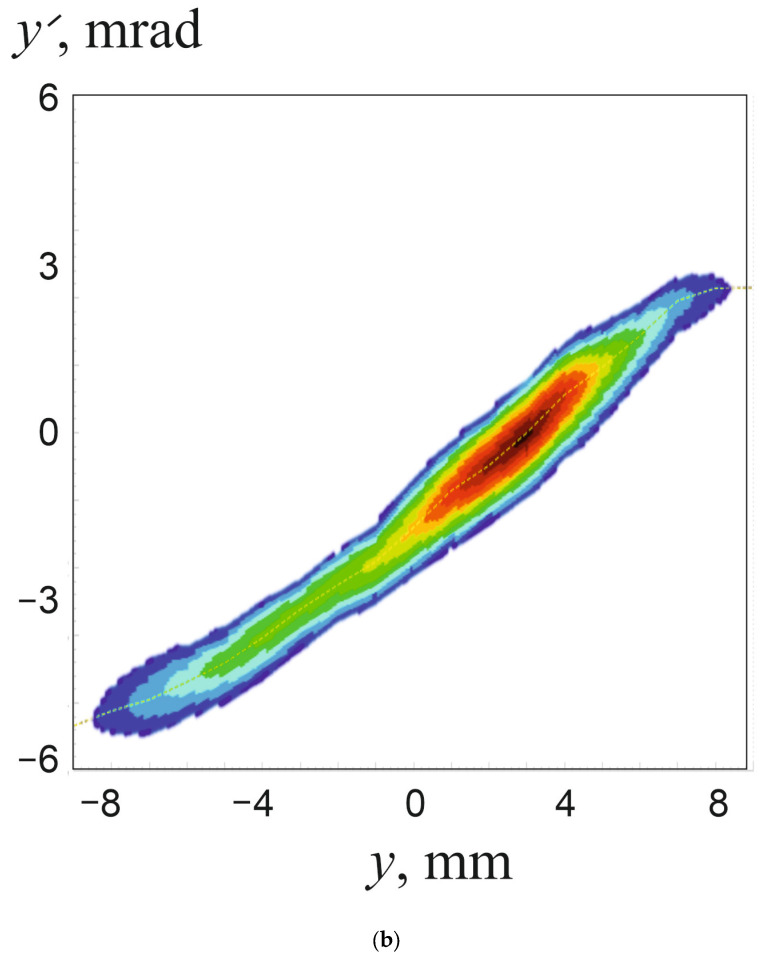
Phase portrait of 3 mA proton beam in VITA facility (**a**) and VITA-II facility (**b**).

The proton beam at the VITA facility has a transverse size of 10 ± 1 mm, angular divergence from ±0.5 mrad to ±1.2 mrad, and normalized emittance of 0.2 mm mrad. The transverse profile of the proton beam is well described by a Gaussian distribution. A typical phase portrait of a proton beam is shown in Figure 8a. An undoubted advantage of such a weakly divergent proton beam is the ability to deliver it to a lithium target without the use of focusing lenses. The only significant disadvantage of this injection mode is the heating of the uncooled diaphragm of the first accelerating electrode.

The proton beam at the VITA-II facility has a transverse size of 15–20 mm, angular divergence from ±3 mrad to ±4 mrad, and normalized emittance of 0.2 mm mrad [32]. The proton beam profile differs from the Gaussian distribution due to spherical aberrations of the pre-accelerator. A typical phase portrait of a proton beam is shown in Figure 8b.

The positive effects of using pre-acceleration are as follows: (i) the proton energy increases by 100 keV and (ii) there is no heating of the uncooled diaphragms of the accelerator due to the smaller size of the ion beam in the accelerator. The negative effects of using pre-acceleration include a deterioration in the quality of the resulting proton beam: it becomes larger, more inhomogeneous, and its divergence increases. We believe that the negative effects outweigh the positive ones. Furthermore, producing such a beam complicates the facility since focusing means are required for its transportation. The use of pre-acceleration itself also complicates the facility—a high-voltage platform and an isolating transformer are required.

To improve the accelerator, we proposed de-accelerating the injected ion beam instead of pre-accelerating it. This was achieved by isolating the input diaphragm of the accelerator and applying a negative potential to it. The implementation of de-acceleration made it possible to reduce the size of the ion beam in the region of the uncooled diaphragm and make the proton beam close to parallel. It is worth noting that significantly slowing down the injected ion beam dramatically improves the stability of the proton beam. The position of the proton beam is virtually independent of the injection angle of the negative hydrogen ion beam. This significantly increases the reliability of the facility.

This result of improving the proton beam due to de-accelerating can be qualitatively explained as follows: slowing down the ion beam into the aperture of the diaphragm due to the negative potential reduces its transverse momentum and promotes stronger subsequent focusing by the input electrostatic lens of the accelerator. It can also be said that the isolated diaphragm under negative potential is an Einzel lens, and its appearance leads to additional adjustable focusing of the beam.

During this study, it was also established that multiple Coulomb (Rutherford) scattering of ions on the atomic nuclei of the stripping gas (argon) in the stripper increases the normalized emittance of the proton beam by an amount comparable to the value of the normalized emittance of the beam of negative hydrogen ions injected into the accelerator, and this process already limits the size of the proton beam.

Thus, in the next version of the VITA facility, we plan to abandon pre-acceleration and implement de-acceleration. To more quickly and reliably obtain the required voltage in the accelerator, we will use seven accelerating gaps instead of six. At the VITA facility, the gas stripper was replaced with a more compact one in 2024 (300 mm long instead of 400 mm, with a 12 mm hole diameter instead of 16 mm) and encountered no problems. Therefore, we will also use a more compact stripper in the VITA-III facility and will be able to maintain the same accelerator size with seven gaps.

### 3.8. Lithium Neutron Capture Therapy

Over the years since the advent of BNCT, the term has become virtually synonymous with NCT. This is partly due to the disappointing results of NCT with gadolinium. However, besides boron and gadolinium, several other elements possess high neutron capture cross-sections, with lithium occupying a special position among them.

The use of lithium instead of boron brings a new, unique opportunity—local 100% energy release since all products of the ^6^Li(n,α)^3^H reaction have high linear energy transfer characteristics. Also, due to the longer range of the ^6^Li(n,α)^3^H reaction products, the requirements for homogeneous intracellular distribution of the neutron capture agent are less stringent. This reaction has remained largely unexplored, primarily due to the previously presumed high toxicity of lithium and the limited availability of lithium-6, which was considered a weapons-grade material.

Taskaeva et al., 2023 [44] marked the beginning of the era of lithium neutron capture therapy. First, we demonstrated in laboratory animals that natural lithium can accumulate in tumor cells to concentrations sufficient for therapy and that its administration at these concentrations does not cause nephrotoxicity. To study lithium nephrotoxicity, male C57BL/6 mice of 10–12 weeks of age with weights of 20–22 g were used. Tumor cells (B16 melanoma) were administered to the animals (1 × 10^6^ cell). After tumor growth was induced, mice were randomly divided into eleven experimental groups (*n* = 5/group): a control group with an intact tumor and groups that were treated by administering lithium carbonate at a single dose of 300 mg/kg or 400 mg/kg orally. The series of experiments for LiNCT was divided into four stages and was carried out using a total of 115 mice with implanted skin melanoma. Mice were sacrificed at 15 min, 30 min, 90 min, 180 min, and 7 days after the start of lithium administration. In the nephrotoxicity assessment experiment, descriptive statistics for lithium biodistribution in blood, tumor and organs, as well as the results of histological examination of the kidneys, are presented in the article by Taskaeva et al., 2023 [44]. After oral administration of lithium carbonate at 300 mg/kg or 400 mg/kg, the peak blood lithium concentration was reached at 90 min: 14.1 µg/mL (≈2.03 mmol/L) and 15.1 µg/mL (≈2.18 mmol/L), respectively. The brain lithium concentration peaked at 4.1 µg/mL (180 min, 300 mg/kg) and 3.1 µg/mL (90 min, 400 mg/kg). Kidney lithium concentrations ranged from 32 to 43 µg/g. Histological examination of the kidneys revealed no signs of lithium-induced nephropathy. No abnormal behavior or weight loss was observed in any of the treated mice. The peak blood lithium level of ≈2.2 mmol/L is below the 2.5 mmol/L threshold for moderate toxicity in humans, and the 24 h half-life of lithium, together with the single-dose protocol, precludes acute toxicity. Thus, this administration protocol achieves therapeutic lithium accumulation without causing acute renal or neurological damage.

Subsequently, for the first time worldwide, lithium neutron capture therapy was performed on tumor-bearing laboratory animals using lithium chloride enriched with a light isotope ^6^Li. To induce tumors, cultured B16 cells were injected subcutaneously into the right inguinal region of mice (2 × 10^6^ cells). After tumor growth induction (10 days in the oral model and 7 days in the intraperitoneal drug administration model), mice were randomly divided into four groups: (1) a control group (intact tumor), (2) a group receiving only lithium, (3) a group receiving only radiation, and (4) a lithium neutron capture therapy group. Animals were randomly assigned to cages on different racks in the same room and maintained under standard laboratory conditions. Irradiation was administered between 1:00 PM and 5:00 PM. Tumor measurements were performed by a researcher blinded to group assignment. Animals were observed until death or euthanasia by cervical dislocation (without anesthesia) in compliance with the approved study protocol. In individual experiments where statistical analysis was conducted, it was performed using *R* language. The normality of the data was evaluated using the Shapiro–Wilk test, with a significance criterion of *p* < 0.05. Tumor volume dynamics were analyzed using a generalized linear mixed-effects model (GLMM). Kaplan–Meier curves were used to evaluate survival times. The log-rank test with Holm’s correction for multiple comparisons was used to estimate *p*-values based on the survival analysis results of the experimental animals. Two-tailed *p* < 0.05 was deemed statistically significant. The results were striking: the treated group showed significantly increased survival and 2–4 times slower tumor growth compared to the three control groups.

Therefore, lithium should be regarded as a valid agent for NCT. Consequently, we call for a reconsideration of the current paradigm that positions boron as the sole agent for this therapy.

## 4. Discussion

The BNCT technique is beginning to enter clinical practice; several clinics are conducting the therapy, several clinics are conducting clinical trials, and several dozen more clinics are in the process of being established [2,3].

To obtain a therapeutic neutron beam in the epithermal energy range, various charged particle accelerators (cyclotrons, linear radiofrequency electrodynamic accelerators, electrostatic accelerators) with proton energies from 2.1 MeV to 30 MeV and various targets (lithium, beryllium) are used. Despite this diversity, “*accelerators producing very different initial neutron source spectra ultimately produce thermal neutron fluences in phantom which have a high degree of similarity*” ([42], p. 5). Further, Green et al., 2025 ([42], p. 6) made a very important statement: “*This promises much for the future of BNCT and will hopefully lead to the possibility of multi-centre trials of this important new cancer treatment technology*”.

Over time, this important new cancer treatment technology will require more accelerator neutron sources, and their efficiency will become more important. As follows from the results of the article by Green et al., 2025 [42], which presents data on the thermal neutron fluence rate of different neutron sources (this can be seen in Figure 8), the use of a lithium target with a proton beam in the region of 2.3–2.5 MeV is more effective than the use of a beryllium target with higher proton energy. Electrostatic accelerators are characterized by greater efficiency than cyclotrons or linear radiofrequency electrodynamic accelerators. For these reasons, a simple, reliable, and efficient VITA-III accelerator neutron source appears to be a good candidate for equipping oncology clinics.

The aforementioned article by Green et al., 2025 [42] also touched on another important aspect: the need to produce a directed neutron beam. The IAEA’s book ([2], p. 27) recommends a beam directivity greater than or equal to 0.7, although in the footnote, it is indicated that for treatment of melanomas, a beam with a “*much lower value (e.g., 0.3) can be used*”. The authors of the article showed “*that higher values (up to 0.87) provide some significant benefit for the introduction of dose verification approaches based on prompt-gamma imaging*”. However, they noted that “*it is feasible to generate a much more directional epithermal neutron beam, but with the cost of a significantly reduced intensity*”. Using the Birmingham facility as an example, they showed that increasing the neutron beam directionality from 0.62 to 0.67 leads to a decrease in fluence to 56%, and an increase in the directionality to 0.87 leads to a decrease in fluence to 26%. We believe the cost of using prompt γ-ray spectroscopy is prohibitively high, so we have proposed and implemented a solution that does not require high neutron beam directionality. Details of the proposal are presented and discussed in Section 3.3.1 and Section 3.6.

We consider prompt γ-ray spectroscopy to be important and reliable, and therefore recommend equipping oncology clinics with it. Its use will allow for adjustments in irradiation timing, if necessary, and will more reliably evaluate treatment outcomes.

Other dosimetry methods are important for characterizing the neutron beam, but ultimately, for therapy, it is crucial to confirm the fast neutron dose, as it alone can vary significantly between different facilities. The other two undesirable doses (thermal neutron dose and γ-ray dose) [36], like the boron dose, are proportional to the thermal neutron flux density, and the only way to suppress them is to increase the ratio of boron concentration in the tumor to boron concentration in the healthy organs. We propose using the “cell dosimeter” [38] for measuring the fast neutron dose. Here, it is important to remember that it is easy to suppress the fast neutron dose by thermalizing the neutron beam, but the depth of neutron penetration (depth of therapy) is reduced.

We believe it is important to study the effects of neutron irradiation on cell cultures and laboratory animals. A number of such studies have been conducted. It is clear that detailed studies of the mechanisms of cell death and changes in the tumor microenvironment are needed. Radiobiological studies are also needed to determine the relative biological effectiveness coefficients and the compound biological effectiveness under conditions typical for clinical use.

The IAEA recommendations [2] are formulated for a neutron beam of the epithermal energy range for the treatment of deep-seated tumors. As we know, an additional moderator is placed between the BSA and the patient when treating superficial tumors at the Kansai BNCT Medical Center (Osaka, Japan). We are currently developing a hydrogen-moderated BSA for the treatment of superficial tumors, which features a higher neutron beam intensity compared to traditional magnesium fluoride-moderated BSAs. In the future, accelerator neutron sources may need to be equipped with two BSAs: one for the treatment of deep-seated tumors and one for the treatment of superficial tumors.

The mechanism of lithium neutron capture therapy (LiNCT) differs fundamentally from BNCT. The ^6^Li(n,α)^3^H reaction releases 4.78 MeV, with both products (tritium and alpha) being high-LET particles. Unlike ^10^B(n,α)^7^Li, where the reaction products have ranges of ~5–9 µm, the tritium from the ^6^Li(n,α)^3^H reaction has a range of ~40 µm in tissue. This longer range relaxes the requirement for homogeneous intracellular drug distribution, which is a known limitation of BNCT when boron delivery is uneven. Consequently, LiNCT may be more effective in tumors with heterogeneous drug uptake. Moreover, 100% of the energy from the ^6^Li reaction is deposited as high-LET radiation, whereas BNCT has a small low-LET component from the 478 keV photon.

Translating LiNCT to clinical practice requires addressing several issues. The therapeutic agent (^6^Li) must be enriched, but current production costs can be comparable to enriched boron. Preclinical data show that a single oral dose of 300–400 mg/kg lithium carbonate yields peak blood levels ≤ 2.2 mmol/L, below the 2.5 mmol/L threshold for moderate toxicity. However, clinical translation possibly will require optimized dosing (intravenous or oral), real-time serum lithium monitoring, and screening for pre-existing renal or thyroid conditions.

Long-term safety for LiNCT concerns potential delayed effects after a single or a few administrations, not chronic toxicity from continuous use (as in psychiatric therapy). In our acute nephrotoxicity studies, no renal damage was observed within 7 days. Given that LiNCT would involve only one or two irradiations, the risk of cumulative toxicity is low, but systematic long-term safety studies are warranted before clinical trials.

Let us compare the VITA facility with other facilities and highlight its advantages and disadvantages. There are five clear advantages to it: (i) high efficiency of conversion of beam power into boron dose rate due to the low energy of neutrons generated in the ^7^Li(p,n)^7^Be reaction; (ii) high efficiency of conversion of electricity into beam power, characteristic of electrostatic accelerators; (iii) the technology for manufacturing an electrostatic accelerator is simpler than that of a radiofrequency accelerator; (iv) compactness; (v) high reliability due to a small number of control devices and implemented solutions, in particular with a lithium target. The neutron beam parameters for epithermal neutron flux density, thermal neutron flux contribution, and contributions of unwanted γ-ray dose and fast neutron dose meet IAEA requirements; the beam directivity parameter is slightly lower than recommended but can be adjusted to comply upon request. Two disadvantages of the VITA facility are related to the use of a lithium target. Due to the inelastic scattering of protons on lithium nuclei, an additional dose is present, but it is small and practically negligible. Neutron generation leads to activation of the lithium target by the radioactive isotope beryllium-7, requiring additional precautions and disposal measures.

In conclusion, we recommend that efforts be made to develop lithium neutron capture therapy, which has several advantages over BNCT, or to develop a combination of the two that can provide a synergistic effect and preserve the imaging method.

## 5. Conclusions

Boron neutron capture therapy is considered a promising method for treating malignant tumors. Currently, several dozen accelerator neutron source projects are being implemented worldwide, with some of them being used for treatment or clinical trials. One of these sources is the VITA^®^ facility, consisting of a tandem electrostatic accelerator of an original design for producing a 2.3 MeV 10 mA proton beam, a lithium target for generating neutrons in the ^7^Li(p,n)^7^Be reaction, and a beam shaping assembly for producing a neutron beam that meets IAEA recommendations.

The article describes the VITA^®^ facilities used for research or clinical trials and presents the results of the studies conducted and the dosimetry tools developed. The possibility of using prompt γ-ray spectroscopy for boron imaging was experimentally demonstrated, its importance was noted, and a recommendation was given to equip oncology clinics with this diagnostic tool for planning therapy and assessing its results. The possibility of implementing lithium neutron capture therapy, which has a number of advantages over BNCT, has been experimentally demonstrated, and its development is recommended, possibly in combination with BNCT, which will allow the preservation of the direct imaging method. This article summarizes the results of several primary studies with laboratory animals and pets, detailed descriptions of which are given in the original publications [35,44].

The VITA^®^ facility is distinguished by its highest efficiency, as shown by a comparison of its thermal neutron fluence rate (boron dose) with other facilities. For this reason, the simple, reliable, and efficient VITA-III accelerator neutron source appears to be a good candidate for equipping oncology clinics. We are interested in manufacturing the VITA^®^ facilities for the purpose of their use in oncology clinics.

The BNCT technique, proposed 90 years ago, is finally entering clinical practice. The scientific community and cancer patients are eagerly awaiting the results of the treatment and the clinical trials currently underway or planned at a number of facilities. Despite the initial neutron spectra of different facilities, the resulting thermal neutron fluxes are highly similar. This opens the possibility of multicenter research and the exchange of obtained information, which is important for the further development of BNCT.

The development of new drugs for boron delivery, which can expand the scope of BNCT or improve it, is becoming a pressing task. Another pressing issue is the development of dosimetry tools, and the prompt γ-ray spectrometry method may be the solution.

Using lithium instead of boron offers several advantages in neutron capture research; therefore, further development is necessary. It should also be noted that lithium, unlike boron, participates in enzymatic intracellular reactions. The use of lithium precludes the implementation of prompt γ-ray spectrometry and requires a new solution.

## 6. Patents

Uspenskij, S.; Haptahanova, P.; Zaboronok, A.; Kupkin, T.; Zeleneckij, A.; Selyanin, M.; Taskaev, S. Method of producing a composition for boron neutron capture therapy of malignant tumors (embodiments). Patent for invention No. 2729458, 30 April 2020 (Russia); Chinese Patent No. CN 114072656 B, 10 March 2023 (China); United States Patent no. US 12,390,527 B2, 19 August 2025 (USA).

Taskaev, S. Method for producing a beam of epithermal neutrons. Patent for invention No. 2722965, 5 June 2020 (Russia).

Taskaeva, I.; Taskaev, S. Method for determining the absorbed dose of recoil nuclei. Patent for invention No. 2743417, 18 February 2021 (Russia).

Taskaev, S.; Makarov, A.; Sokolova, E. Systems, devices, and methods for deformation reduction and resistance in metallic bodies. United States Patent no. US 2022/0030696 A1, 27 January 2022 (USA); Patent for invention RU 2825858 C2, 2 September 2024 (Russia).

Kolesnikov, Ya.; Taskaev, S.; Savinov, S.; Bykov, T. Device for measuring the phase portrait of a powerful stationary beam. Patent for invention No. 2839258, 28 April 2025 (Russia).

Berendeev, E.; Gorlachev, G.; Shein, T.; Koshkarev, A.; Koshechkin, S.; Taskaev, S. VITA Dosimetric Planning System. Certificate of State Registration of Computer Program No. 2025685234, 19 September 2025 (Russia).

Berendeev, E.; Koshechkin, S.; Koshkarev, A.; Shein, T.; Taskaev, S. Method for planning boron neutron capture therapy and evaluating its results. Patent for invention RU 2850118, 5 November 2025 (Russia).

Kasatov, D.; Konovalova, V.; Taskaev, S. Method for measuring the 478-keV line detection efficiency of a gamma spectrometer. Patent No. 2850742, 13 November 2025 (Russia).

Kolesnikov, Ya.; Ostreinov, G.; Savinov, S.; Taskaev. S. Charged particle accelerator. Patent for invention No. 2852336, 8 December 2025 (Russia).

## Data Availability

The data presented in this study are available on request from the corresponding author.

## Abbreviations

The following abbreviations are used in this manuscript:

BPA	boronophenylalanine
BSA	beam shaping assembly
BSH	sodium borocaptate
BINP	Budker Institute of Nuclear Physics (Novosibirsk, Russia)
BNCT	boron neutron capture therapy
HDPE	high-density polyethylene
LiNCT	lithium neutron capture therapy
NCT	neutron capture therapy
PMMA	polymethyl methacrylate
TPS	treatment planning system
VITA	(1) Vacuum-Insulated Tandem Accelerator (2) A trademark for the VITA accelerator neutron source, its components, software, and diagnostic equipment. Applies to physics research, medical research, clinical trials, and materials testing. The trademark was registered on 19 May 2026, no. 1222655.
